# Confounds in “Failed” Replications

**DOI:** 10.3389/fpsyg.2019.01884

**Published:** 2019-09-04

**Authors:** Paola Bressan

**Affiliations:** Dipartimento di Psicologia Generale, University of Padova, Padova, Italy

**Keywords:** replication, confounds, good research practices, Open Science Collaboration, reproducibility project, mate preferences, ovulatory shift

## Abstract

Reproducibility is essential to science, yet a distressingly large number of research findings do not seem to replicate. Here I discuss one underappreciated reason for this state of affairs. I make my case by noting that, due to artifacts, several of the replication failures of the vastly advertised Open Science Collaboration’s *Reproducibility Project: Psychology* turned out to be invalid. Although these artifacts would have been obvious on perusal of the data, such perusal was deemed undesirable because of its *post hoc* nature and was left out. However, while data do not lie, unforeseen confounds can render them unable to speak to the question of interest. I look further into one unusual case in which a major artifact could be removed statistically—the nonreplication of the effect of fertility on partnered women’s preference for single over attached men. I show that the “failed replication” datasets contain a gross bias in stimulus allocation which is absent in the original dataset; controlling for it replicates the original study’s main finding. I conclude that, before being used to make a scientific point, all data should undergo a minimal quality control—a provision, it appears, not always required of those collected for purpose of replication. Because unexpected confounds and biases can be laid bare only after the fact, we must get over our understandable reluctance to engage in anything *post hoc*. The reproach attached to *p*-hacking cannot exempt us from the obligation to (openly) take a good look at our data.

*Examine [the data] from every angle*.

Daryl J. Bem (1987, p. 172)

## Introduction

Reproducibility may be crucial in science, but originality presents itself better. Thus, the activity of merely reproducing the work of others has been infrequent (Makel et al., 2012) and regarded with contempt. The spirit of the times has now briskly turned. We are in the midst of a movement that attaches increasing importance to repeating original studies—while loudly questioning the credibility of those findings that do not appear to replicate.

Yet the idea that we should trust a failed replication more than the original study is debatable. A failed replication—unless it has higher statistical power (Maxwell et al., 2015) or does a better job of meeting some implicit auxiliary assumption linking theory to observation (Trafimow and Earp, 2016)—is bound to be just as unreliable as the study it fails to replicate. An effect that truly exists in the world will not always prove “statistically significant” in a faithful replication of the original study; the *p* values produced by repeated simulations of the same experiment bounce around to a rather alarming extent (“the dance of the *p* values”: Cumming, 2014; see also Stanley and Spence, 2014; Van Calster et al., 2018). That people would expect *p* values to stay put, naturally, scarcely helps them grasp what nonreplications (do not) entail—reinforcing the feeling of a replication “crisis” (Amrhein et al., 2019).

In this article I illustrate a complementary reason for being skeptical of failed replications: the effect may be there, but remain unseen due to the authors’ well-meant unwillingness to treat the new data any differently than the original ones. The wholly understandable aversion to engaging in *post hoc* practices appears to have gone overboard. It is currently feeding the argument that, because “any well-designed study (e.g., an adequately powered study with appropriate measures) provides useful information regardless of the specific findings” (Johnson et al., 2014, p. 320), peer review is only needed before, and not after, data collection. Alas, the property of coming from a well-designed study does not automatically endow data with the distinction of providing useful information. Not only can an “adequately powered study with appropriate measures” produce nonsense, but crucially, there is no knowing ahead of time whether and how it will. We find out if something went *unexpectedly* wrong only by looking at the data (assuming, that is, we are lucky and the data will tell).

## The Million Roads to the Null Effect

I shall illustrate my point with actual cases taken from the *Reproducibility Project: Psychology* (Open Science Collaboration, 2015), whose results made it into *Science* and proceeded to gather nearly 3,000 citations in 3 years. This project attempted to replicate 100 studies published in 2008 in three respected psychology journals: *Psychological Science*, *Journal of Personality and Social Psychology*, and *Journal of Experimental Psychology: Learning, Memory, and Cognition*. Of the original studies whose results were significant, slightly over 60% failed to replicate—that is, yielded nonsignificant results (*p* ≥ 0.05).

Here I showcase a few of the nonreplications that, unbeknownst to the public, turned out to be invalid. I am including only cases in which something amiss was found in the data themselves—rather than, or besides, the methods or analyses—and a response was written up about it. The problems were invariably caught by the original authors; links to each replication report and original authors’ response are presented along the original article’s reference, in the References list. Most such responses have been added to the corresponding replication record on the Open Science Framework platform. Still, they have not prompted corrections or updates to the replication’s status (such as replacing “failed” with “invalid” or “inconclusive”) and do not appear to do much else than sit there.

### “Failed” Replication of Amodio, Devine, and Harmon-Jones (2008)

In a racial-stereotype inhibition task, people with low levels of prejudice did better when their motivations were only internal (e.g., when being nonprejudiced was personally important to them) rather than external too (e.g., when appearing nonprejudiced served to avoid disapproval from others) (Amodio et al., 2008). This more efficient inhibition of racial stereotypes reflected better stereotype control specifically, as opposed to better cognitive control in general.

The well-validated task used here to measure stereotype control consists in having people classify images of pistols, drills, and suchlike as either guns or tools. Right after seeing very briefly a Black (as opposed to White) face, people are more likely to classify correctly a gun than a tool; the larger one’s tendency to do so, the weaker one’s stereotype control is surmised to be. This normally observed effect was missing entirely in the replication data, rendering the task invalid as a measuring device. One possible reason is that, although the point was to examine Whites’ racial biases toward Blacks, and the original sample included primarily White participants, the majority of participants in the replication sample turned out to be non-White.

### “Failed” Replication of Campbell and Robert (2008)

In a practice phase, people repeatedly solved both multiplication problems (such as 7 × 5 = ?) and factoring problems (such as 6 = ? x ?) (Campbell and Robert, 2008). In the test phase, half of the participants were only asked to multiply and the other half to factor. People who were asked to multiply were faster at solving the problems they had previously practiced as multiplications (7 × 5 = ?) than those they had practiced as factoring (3 × 2 = ?). However, they were faster at problems they had practiced as factoring than at new multiplications altogether. The same result, in reverse, held for those who were asked to factor. Thus, although people did best with problems identical to those practiced earlier (as one would expect), cross-operation transfer was observed too; this was the important result.

In the replication, no evidence of transfer between multiplying and factoring was found. Curiously enough, in the Reproducibility Project database this replication is marked as successful—on the grounds that the significant interaction found in the original study was significant here too. This was a mistake, because (as also pointed out in the replication report itself) the data patterns that produced the interaction were different in the original and replication studies: a practice effect plus a cross-operation transfer in the original, just a practice effect in the replication.

Inspection of the replication data showed that participants failed to become much faster with practice, and during the practice phase continued to make a lot of errors (which, there being no feedback, remained uncorrected and hence did not promote learning). After the entire set of 20 blocks of practice of the same eight problems, one of the two groups was still making 10 times as many errors as the corresponding group in the original study. Surely, one cannot expect much transfer of something that has not been learned in the first place.

### “Failed” Replication of Monin, Sawyer, and Marquez (2008)

Moral rebels are individuals who refuse to comply when complying would compromise their values. According to Monin et al. (2008), people who do comply dislike rebels because their own obedient behavior is implicitly called into question by the rebel’s behavior, and this threatens their self-confidence. If so, buttressing people’s self-confidence should reduce their need to disparage moral rebels. Here, participants who had just completed a self-affirmation task (i.e., written an essay about a recent experience in which they demonstrated a quality that made them feel good about themselves) disliked moral rebels less than did participants who had completed a control task instead (i.e., listed foods consumed in the last 24 hours).

The crucial manipulation consisted in having participants write a long, mindful essay aimed at increasing their sense of being a good, worthy person. In the original study, this was an 8-minute composition written in the laboratory; the median number of words was 112. In the replication study, which was online, the amount of time participants were required to spend on the essay was not specified; the median number of words turned out to be 29, suggesting that most people had just rushed through the task. On top of that, being Monin et al.’s article about rebels’ rejection by their peers, the target person portrayed as either a complier or a rebel ought to have been a peer: in the original study, it was a fellow student of the same age. However, the replication data revealed a median age difference of 15 years between participant and “peer” (consistent with this reduced similarity, the “peer” was liked less overall). I would personally add that, not coming from a peer, the target person’s behavior might also have felt less directly relevant to the participant’s self-image: less supportive in case of a complier, less threatening in case of a rebel.

### “Failed” Replication of Schnall, Benton, and Harvey (2008)

In this study, participants were asked to judge the morality of hypothetical actions (for example, how wrong it would be to put false information on a résumé) (Schnall et al., 2008). People who had previously been exposed to words related to purity and cleanliness (or had washed their hands after watching a disgusting film clip) made more lenient moral judgments than people who had been exposed to neutral words (or had not washed their hands).

Perusal of the replication data disclosed that, across the various moral scenarios, a large percentage of responses was at the top of the scale (“extremely wrong”: 41% vs. 28% in the original study). This implies that the lack of effect in the replication study may have resulted purely from lack of variance due to a ceiling effect (Schnall, 2014a,b). (But see Huang, 2014, for discussion of another variable—replication participants’ low vs. high response effort—which would appear to be more critical.) The replicators downplayed Schnall’s concerns on the grounds that “the distributions themselves provide valuable information for the field about the generalizability of the original findings” (Johnson et al., 2014, p. 320), but this is true only in a loose, uninteresting sense. The specific information they provide is that the original moral scenarios are sensitive to changes in context. They say nothing about the original findings themselves—which, with moral scenarios better suited to the replication sample (i.e., permitting as much variance as in the original study), could replicate just fine.

## The Best Men are (Not Always) Already Taken

In each of the cases just reviewed, the replication data were unable to speak to the question of interest and it was too late to do something about it. Amodio, Campbell, Monin, and Schnall had no way of showing that the failed replication would have been successful had the confounds not been there. The causal link between confounding variables and null effects was suspected but not proven.

To make a stronger argument, let us look again at the Reproducibility Project’s original studies that failed to replicate. Here I pick yet another such study (Bressan and Stranieri, 2008) that appeared in *Psychological Science* and that I happen to know especially well, being its senior author. The reason why this case deserves closer attention than the others do is that, remarkably and uncommonly, some unconfounding of the confounded replication data turned out to be possible.

The study found that women’s preference for faces of men described as single, relative to faces of men described as attached, depended on the ovulatory cycle. Higher-fertility women (those in the middle 2 weeks of their monthly cycle) preferred single men more than did lower-fertility women (those in the first and last week of their cycle). A significant interaction between fertility and women’s relationship status indicated that the effect was specific to women who had a partner.

Bressan and Stranieri (2008) pointed out that the effect was consistent with the hypothesis of female dual mating (Pillsworth and Haselton, 2006; see also Thornhill and Gangestad, 2008; Gildersleeve et al., 2014). Over evolutionary history, some women may have benefitted from having their long-term partner raise a child they had conceived with a more attractive man. If the children of these arrangements turned out to be reproductively more successful than the children of women who never strayed (whatever their circumstances), this adaptation would have spread.

Note that the implication of this hypothesis is not that women gain from seeking extrapair partners—only a minority of women in a minority of circumstances would (e.g., Buss and Shackelford, 2008). The implication is, instead, that women have evolved to be able to flexibly implement this strategy *should* they find themselves in these particular circumstances. Indeed—not on moral, but on evolutionary grounds—extrapair mating ought not be pursued liberally. First, sex, especially with a stranger, invariably involves the risk of infection or injury. Second, female adultery is punished, often harshly, in virtually every society (Buss, 2000). It follows that an adaptation to stray could have evolved only if the hazard brought fruit often enough.

Women, then, might be hardwired to find men more attractive when the odds of conceiving are higher rather than lower. Several lines of evidence suggest that indeed they do: being in the fertile window increases sexual desire for extrapair partners (e.g., Gangestad et al., 2002; Arslan et al., 2018; as can be seen by comparing these two works, evidence of whether this shift extends to in-pair partners is mixed). Single men are more available as extrapair partners than are already attached men. Thus, the effect of fertility on women’s preference for single (over attached) men may be an adaptation that increases the benefits of adultery over its costs (Bressan and Stranieri, 2008).

In this article, I am not concerned with “defending” the hypotheses discussed by Bressan and Stranieri (2008); they are just hypotheses. What I care about is whether the main finding is replicable. The Reproducibility Project failed to replicate it across two experiments, one conducted in the laboratory and one online (Frazier and Hasselman, 2016). Although some minor results were replicated, no effect whatsoever of the ovulatory cycle on women’s preferences for single men was found. Here I reanalyze these data and show that they contain unforeseen confounds that were absent in the original dataset. Once these confounds are controlled for, the data reveal the same pattern as those in the original study.

## Methodological Matters

### Participants

**Figure 1:**
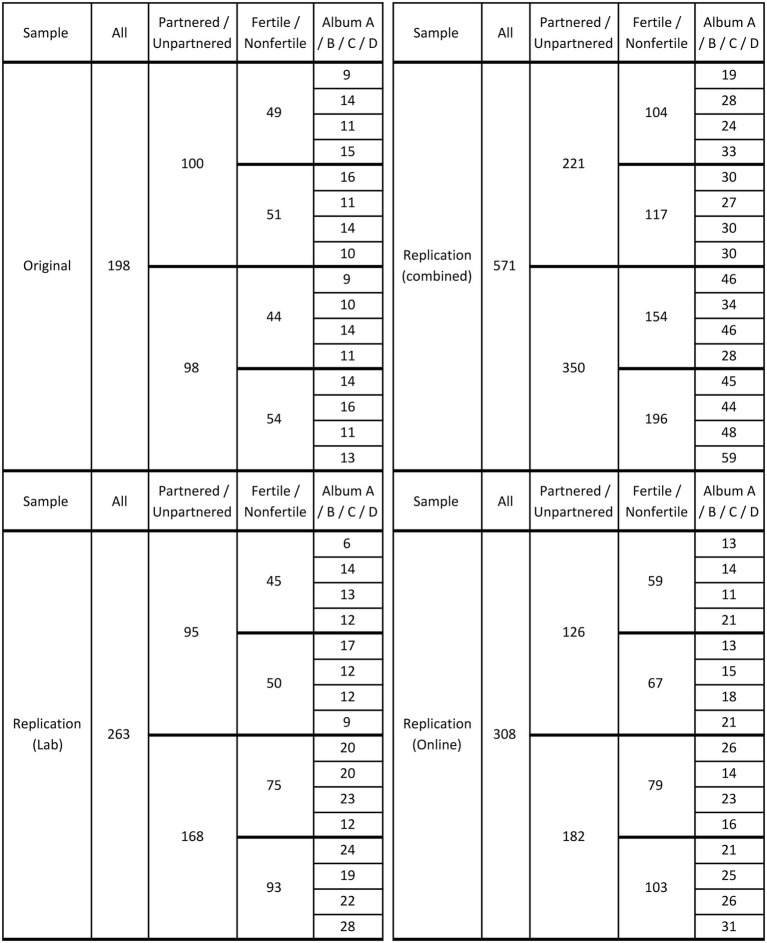
Number of partnered/unpartnered, fertile/nonfertile women who participated in the original (top-left panel), lab replication (bottom-left panel), and online replication (bottom-right panel) studies. The top-right panel presents the combined replication data, which along with the original data were used for my reanalyses. Each participant was shown one photo album out of four possible ones; the distribution of the four albums (A, B, C, D) across participants is indicated in the last column of each panel.

A total of 769 heterosexual, normally cycling women were included in the analyses (Figure 1). Original sample—Italian (Bressan and Stranieri, 2008): Italian ethnicity, median age 21 years, range 18–35. Lab replication sample—American (Frazier and Hasselman, 2016): mixed ethnicities, median age 18 years, range 16–46. Online replication sample—American (Frazier and Hasselman, 2016): mixed ethnicities, median age 21 years, range 18–34.

Participants’ eligibility criteria, along with the manner variables were coded, were identical for the original and replication datasets and were the same as in Bressan and Stranieri (2008). In all datasets, each woman’s cycle day had been standardized by dividing the number of days since the first day of her last menstrual period by her reported typical cycle length and multiplying the quotient by 28. Based on this index, women had been divided into a higher-conception-risk group (days 8–20) and a lower-conception-risk group (days 1–7 and 21–28). This subdivision (the “average midcycle rule”: Lamprecht and Grummer-Strawn, 1996) has the advantage of creating two approximately equal groups. Note that, in Bressan and Stranieri’s original dataset, standardized cycle days had been rounded to the nearest integer, so that, for example, a participant on day 7.7 (which rounds to 8) would be in the high-conception-risk group. In both replication datasets, on the opposite, it appears that standardized cycle days had not been rounded, so that a participant on day 7.7 would be in the low-conception-risk group. To render the data comparable, I adopted the least disruptive, most conservative choice, and avoided rounding in both the original and replication datasets. This reclassified as nonfertile four original-study participants (one partnered, three unpartnered) that had been treated as fertile in Bressan and Stranieri (2008). So, all and only women on days 8.0–20.0 were labeled as fertile (high-conception-risk group) in all three datasets.

Women who were taking hormonal contraceptives, were on a standardized cycle day larger than 28 (i.e., experiencing an abnormal ovulatory cycle), reported not being heterosexual, or failed to disclose their relationship status were excluded from all datasets; all other participants were included. In both datasets provided by the Reproducibility Project team (see Data Availability), exclusions had already been made. The lab replication dataset was used as is. The online replication dataset revealed errors in the calculation of women’s cycle day; correcting them removed seven participants (see section “Coding errors in the replication datasets” for details). Note, however, that these corrections did not affect the results.

### Stimuli

Twelve color photographs of faces of men of various degrees of attractiveness were presented in an album, one per page. Each photo was accompanied by one of four labels: “this person is single,” “this person is in love,” “this person has a girlfriend,” and “this person is married.” Four parallel albums were prepared so that each of the 12 faces could be paired, between subjects, with all four labels. Stimuli and albums were the same across the original and replication studies (see Data Availability). Stimuli were presented on paper in the original and lab replication studies, on a computer screen in the online replication study.

### Procedure

In the original study, participants were asked to imagine being at a party (with their partner, if they had one) and seeing the man portrayed in the photograph. They read aloud the accompanying label and then rated the man’s attractiveness on a scale from 0 (not at all attractive) to 10 (very attractive). The lab replication followed a similar procedure. The online replication’s method was adapted to the different interface, and included a memory test for each face/label combination to make sure that the label had been read.

In the original study, after going through the photos, participants answered several questions (some of which were meant to provide information for an unrelated study on female competition) about themselves and their partner, if they had one. The original questionnaire was in Italian; replication participants filled in the exact same questionnaire in an English translation (see Data Availability).

## Looking at the Data

Inspection of the replication report (Frazier and Hasselman, 2016) and datasets (Data Availability) uncovered reporting errors in the analyses (one of omission, one of commission), coding errors in the dataset, and sources of random and of systematic noise (confounds). Yet it is important to note that it was the confounds, not the errors, that were responsible for the failure to replicate.

### Reporting Errors in the Replication Analyses

Following Bressan and Stranieri (2008), the replication team averaged the attractiveness ratings for the three categories of attached men (married, with a girlfriend, and in love) for each participant. The preference for single men was computed as the mean rating given to single men minus the mean rating given to attached men. These measures had already been calculated for both replication datasets, and in my reanalyses I used them exactly as they appear in the Reproducibility Project’s files (Data Availability).

The replication authors reported (Frazier and Hasselman, 2016) that, unlike in Bressan and Stranieri’s original study, the interaction between man’s availability (single, attached), participant’s conception risk (low, high), and participant’s partnership status (partnered, unpartnered) was not significant (*F* < 1 in both the lab and online replications; repeated-measures ANOVAs). I reran their analyses on exactly the same data and in exactly the same way.

The lab replication analysis came out identical. In the online replication analysis report I found one error (surely a typo) and one remarkable omission. Along with the critical triple interaction (*p* = 0.746), the authors reported the following effects: partnership status (*p* = 0.008), conception risk (*p* = 0.548), and man’s availability (*p* = 0.091; the corresponding *F* was misreported as 16.90 whereas it should have been 2.88). This list of significant and nonsignificant effects failed, however, to include the nearly significant interaction between conception risk and man’s availability: *F*(1, 314) = 3.71, *p* = 0.055. As shown by separate ANOVAs, this interaction was due to the fact that fertile women liked single men better than attached men, *F*(1, 139) = 6.62, *p* = 0.011, whereas nonfertile women did not, *F* < 1. This effect is in the same direction as that found in the original study (where it was further qualified by the interaction with partnership status).

### Coding Errors in the Replication Dataset

Inspection of the online replication data file (Data Availability) uncovered a systematic error in the calculation of women’s cycle day. Day 1 (referring to participants on their first day of menstruation) had been miscoded as Day 0 and so on, so that all cycle-day values were off by 1. Correcting these data led to the reassignment of seven low-fertility women to the high-fertility group and of eight high-fertility women to the low-fertility group, and to the loss from the database of six women whose standardized cycle day of 28 turned out to be 29 (meaning that they were experiencing an abnormal ovulatory cycle). One further inclusion error was found: one participant had been assigned a negative cycle day, because the first day of her last menstrual period had been set in the future. Note, however, that neither the correction of the cycle-day values nor the removal of these seven participants had any bearing on the results.

### Sources of Random Noise in the Replication Dataset

Inspection of the lab replication data file (Data Availability) revealed that: (1) 16 participants “arrived late/early, did not follow instructions, had previous knowledge of the study, etc”; (2) 39 participants “forgot to read labels, misread labels, gave ratings before reading labels, questioned labels, asked explicitly whether label should affect her rating”; and (3) 41 participants were “not paying attention, went through very fast, phone usage.” None of these participants (89 overall, because a few fell in more than one category) had been excluded from the analyses run by the replication team. These sources of noise in the data (absent in the original study) were indeed hard to remove, because the study’s statistical power would decrease substantially by dropping these participants en masse^1^. Given the arbitrariness of any decisions about which cases to exclude and which to include, I discarded none of them from my reanalyses either.

Some of the participants in the replication studies had given abnormally low ratings to their current partner’s personality; these women may have been more interested in replacing him altogether than in having him raise their child. However, given that no outlier exclusions based on partner traits had been made by Bressan and Stranieri (2008), I did not make any in my present analyses of the replication data either. Note that the conception-risk effect reported below does become stronger if these outliers are removed; but not being the focus of the current paper, here this point is neither spelled out nor discussed further.

### Sources of Systematic Noise in the Replication Dataset

#### Confounds: Album

Before rerunning the analyses on the corrected replication data, I checked for any relevant differences between the original and replication samples. I began by examining the distribution of the four albums across participants. Different participants saw different albums (with one-fourth of each sample’s participants sharing a specific assortment of face/label combinations). However, because the 12 men whose pictures were used as stimuli had been deliberately chosen so as to cover different degrees of attractiveness (see Bressan and Stranieri, 2008), the three specific men labeled as “single” in each of the four albums were not equally attractive across albums. Hence, the choice of counterbalancing the face/label combinations represented an inevitable source of noise. Album had indeed a significant main effect on the preference for single men in all three datasets. Combining the datasets revealed a large overall preference for singles in two albums (A and C; both *p’*s < 0.0001, one-sample *t*), a large overall preference for attached men in another (B; *p* < 0.0001), and no significant preferences in the remaining album (D; *p* = 0.173). (The pattern of these preferences across albums was the same for partnered and unpartnered women).

In the original study, album did not interact with any of the other variables and contributed random noise only. In the replication study, however—presumably due to some quirk in the recruitment of participants—albums were not uniformly distributed across the various categories of relationship status and conception risk. Crucially (see top-right panel in Figure 1), the two albums with the most attractive single men turned out to have been overwhelmingly presented to fertile women who were unpartnered (unpartnered: 92; partnered: 43; *X^2^* = 8.42, *p* = 0.004; the corresponding figure for the original study is *X^2^* = 0.47, *p* = 0.493), while the two albums with the least attractive singles were presented to equivalent numbers of unpartnered (62) and partnered (61) fertile women. Put differently, the least attractive single men had been shown more often to nonfertile (103) than to fertile (62) unpartnered women, whereas the most attractive singles had been shown to equivalent numbers of nonfertile (93) and fertile (92) unpartnered women.

The original study found that fertile *partnered* women preferred singles. The figures above show that, in the replication study, fertile *unpartnered* women got—by some turn of chance—to rate the best singles. This confound had the remarkable consequence of creating a spurious “fertility effect” for unpartnered women, in the same direction as the original fertility effect for partnered women. Therefore, it made it impossible to detect the original study’s interaction between fertility and relationship status.

It appears indisputable, at this point, that any analysis of the *replication* data that addresses the effects of fertility and relationship status on preference for singles while neglecting the confound of album assignment is bound to deliver noise as an answer. Therefore, I kept track of the effect of album in all analyses (including, as a robustness check, the reanalyses of the original data).

#### Potential Confounds: Self-Confidence With Men

**Figure 2:**
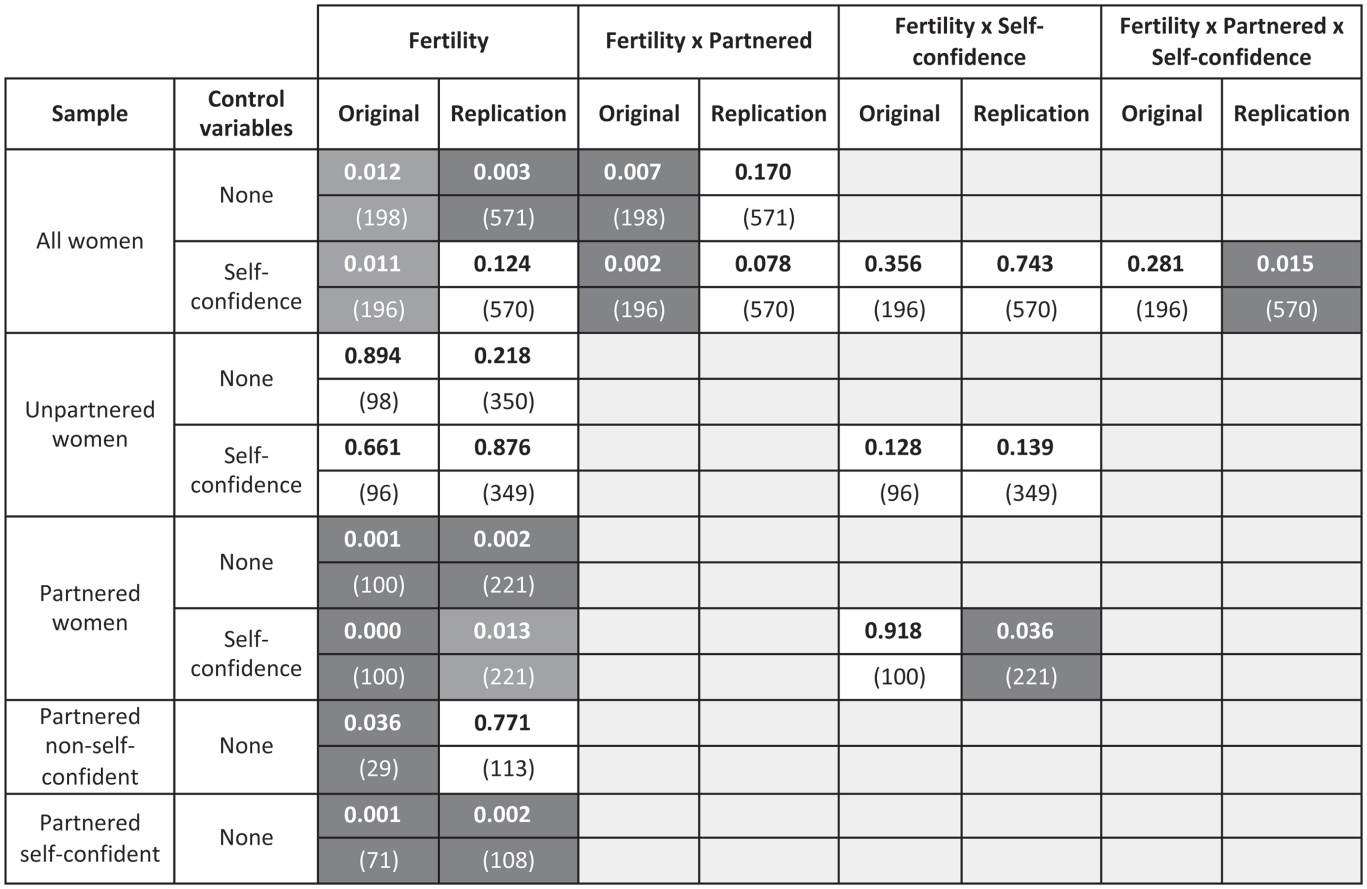
Visualization of the effect of fertility on women’s preference for single relative to attached men. The figure presents the results of univariate ANOVAs; fertility is reported as a main effect and in its interaction with relationship status and/or self-confidence with men, whenever either factor appears in the analysis. The column “Control variables” indicates whether the ANOVA contains factors other than type of study, album, relationship status, and fertility. For each analysis and effect, the cell indicates the *p* value (rounded to the first three digits; *N* within brackets), separately for the original and the replication data. Significant results (*p* < 0.05) are shown in white on a dark background. Significant main effects that are qualified by an interaction are shown on a lighter gray background. Nonsignificant effects are shown in black on a white background.

As mentioned earlier, the replication team did find (though it failed to report) a nearly significant interaction between women’s conception risk and man’s availability. Yet, unlike in the original study, these two factors did not participate in a triple interaction with women’s partnership status. Once one simply controls for the bias in album assignments, as we will see, the interaction with partnership status becomes *p* = 0.170 (Figure 2), raising the question of whether it may have reached significance if only the replication study had been less messy. However, let us assume that the lack of interaction in the replication is to be taken at face value. The first point that comes to mind is then whether partnership status might have affected the American and Italian samples’ women in different ways. In an exploratory rather than confirmatory spirit, I investigated this issue by using the participants’ responses to the questionnaire (see Data Availability). Partnered and unpartnered women saw two different versions of the questionnaire; any shared questions about the partner referred to the current partner in the former case and to a hypothetical partner in the latter. I considered the only question that had been presented identically, and with the same meaning, to both partnered and unpartnered women. This was: “In relationships with the opposite sex, how self-confident are you?” Responses were given on a 1–5 scale (1 = not at all, 2 = a little, 3 = moderately, 4 = a lot, 5 = very much).

The distribution of these responses differed significantly between the original and replication datasets (Mann-Whitney U test, *p* = 0.001). Over 30% of women in either replication sample reported being more than moderately self-confident with men (responses 4 and 5: “a lot” and “very much”), as opposed to less than 20% of women in the original sample (only responses 4; nobody chose the value 5). Critically, in the replication data the distribution of responses was consistently different for partnered and unpartnered women (Mann-Whitney U test, *p* < 0.0005 in both replication samples), unlike in the original data (Mann-Whitney U test, *p* = 0.587). Basically, in American women self-confidence with men was strongly associated with relationship status, being lower for unpartnered than for partnered women. This was not the case in Italian women, which might reflect a general cultural difference or merely a sample difference.

This divergence between the original and replication studies was especially disturbing because in the replication data, unlike in the original data, participants’ self-confidence with men interacted not only with relationship status, as mentioned above, but also with conception risk and album. Strikingly, for example, among extremely self-confident partnered women (response 5) the albums with the most attractive single men had been shown nearly exclusively to those who were nonfertile (nonfertile: 14; fertile: 1), while the least attractive singles were presented to equivalent numbers of nonfertile and fertile women (nonfertile: 10; fertile: 9).

Because a woman’s self-confidence with men is likely to increase the extent to which she perceives a man to be available to her, this set of asymmetries created a serious potential confound that was absent in the original data. For this reason, data were analyzed both with and without considering participants’ self-confidence with men—median-split into “low” and “high”^2^—as a factor in the ANOVA. As a robustness check, this was done for both the original and replication data.

Note that this confound oddly complements and compounds those identified previously. In sum, albums were poorly allocated across (1) partnered and unpartnered fertile women; (2) fertile and nonfertile unpartnered women; (3) fertile and nonfertile self-confident partnered women. All misallocations tended to spuriously increase the ratings given to single men by unpartnered fertile women and/or decrease the ratings given to single men by the most self-confident partnered fertile women. Thus, each confound biased the data in the same direction—opposite to the original result.

## “Failed Replication” Data Reanalysis

### Results

What is at issue here is not how much confidence we should place in the original finding, but whether the Reproducibility Project did indeed, as claimed, fail to replicate it. Hence, I will not be evaluating the magnitude of the effect, the strength of the evidence for it, or the likelihood that the hypothesis is “true”—these matters are all beside the point. Instead, I will be running the very same analyses, only correcting for confounds, and adopting the very same rules and statistical standards as the Reproducibility Project did—whether or not these are the wisest. And because the criterion used to judge success or failure in the Reproducibility Project replications was the presence or absence of statistical significance, this is the criterion I will use, too.

The main analysis reported in Bressan and Stranieri’s original study was a repeated-measures ANOVA on attractiveness ratings with a within-subjects factor of man’s availability (single, attached). For simplicity, it is replaced here with a univariate ANOVA on preferences for single men relative to attached men; the two analyses (repeated-measures on a two-level within-subject variable and univariate on the difference between such levels) are conceptually identical and give identical results.

The fixed factors were album (A, B, C, D), participant’s partnership status (partnered, unpartnered), and participant’s conception risk (low, high). The same univariate ANOVA was run on both the original data and the combined replication data (see text footnote 1). In the latter, type of study (lab replication, online replication) was also added as a factor. Interactions were explored by stratifying the data (by partnership status, as in Bressan and Stranieri (2008), whenever this variable participated in the interaction) and repeating the ANOVA within each subgoup. All ANOVAs were run with and without the potential confounder of self-confidence with men (below the median, above the median) as an additional fixed factor.

For reasons of transparency and completeness of information, main and interaction effects that were significant in one sample and not in the other were explored in both, and all results are reported^3^. Figure 2 presents the *p* values of all effects, separately for the original and replication studies.

Conception risk was significant as a main effect in both the original^4^ and replication data (Figure 2, row 1: compare cells 1 and 2). Overall, higher-fertility women preferred single over attached men more than did lower-fertility women. In the original sample, the main effect of fertility was qualified by a significant interaction with partnership status, whether or not self-confidence with men was added to the analysis (row 1, cell 3; row 3, cell 3). In the replication sample, the main effect of fertility was qualified by a significant interaction with partnership status *and* self-confidence with men (row 3, cell 8).

**Figure 3:**
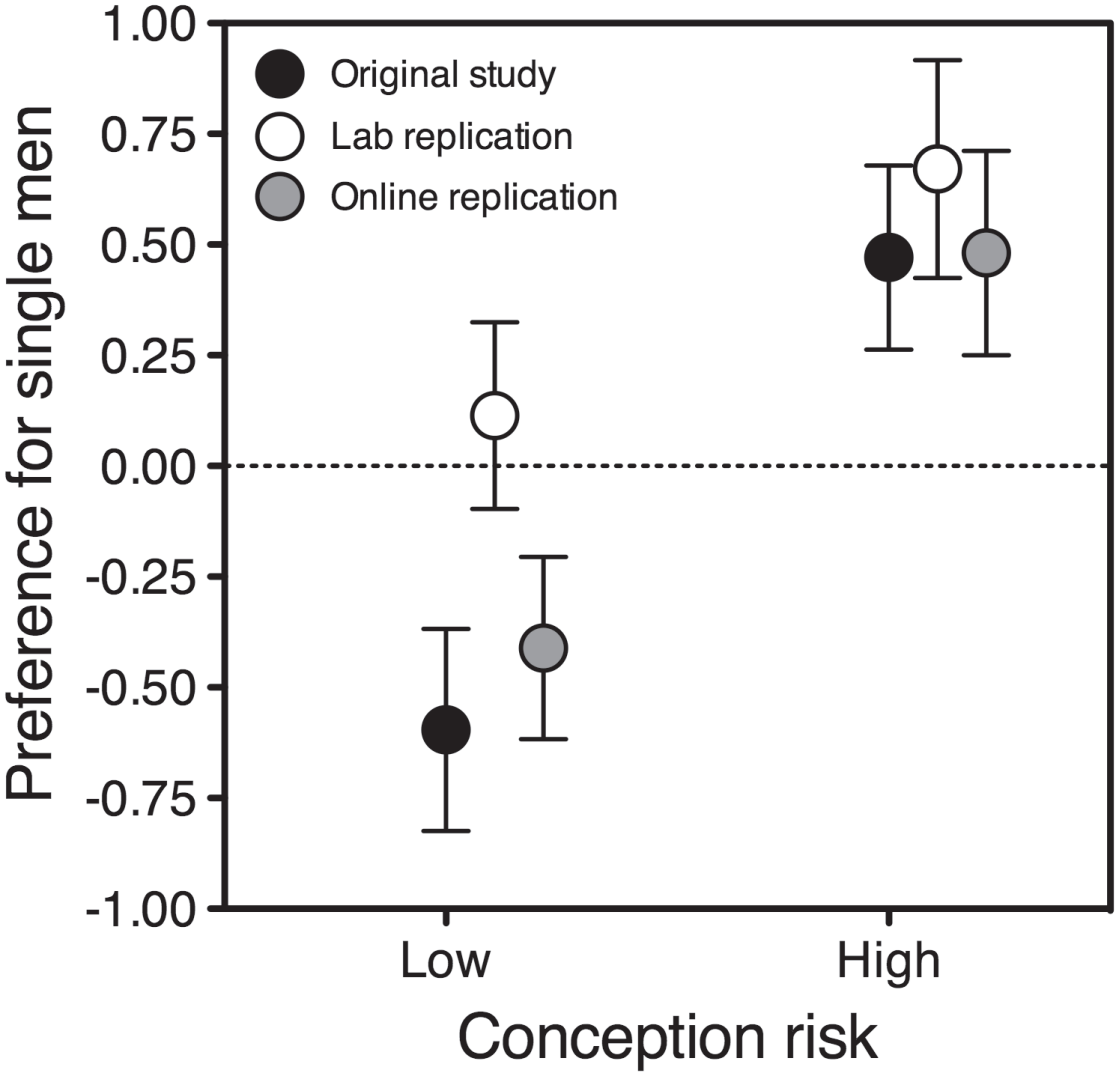
Preference for single over attached men as a function of conception risk, in partnered women who reported above-the-median self-confidence with men. The ovulatory shift is represented by the difference between each symbol on the left and the corresponding symbol on the right. Symbols depict estimated marginal means adjusted for the effect of album; error bars indicate the standard error of the mean. Black symbols: original study, *N =* 71; gray symbols: online replication, *N =* 62; white symbols: lab replication, *N =* 46.

To unpack these interactions, data were stratified by partnership status and fed into separate ANOVAs. In partnered women, fertility was always significant whether or not self-confidence with men was taken into account (rows 9 and 11: compare cells 1 and 2); in unpartnered women, it never was (rows 5 and 7: compare cells 1 and 2). This was true in both the original and replication studies. In the latter, the significant effect of fertility in partnered women was further qualified by a significant interaction with self-confidence with men (row 11, cell 6). Exploring this interaction showed that the effect was entirely driven by self-confident women (row 15, cell 2; see Figure 3).

### Discussion

Failures to replicate can certainly suggest that the original findings emerged by chance, but we should contemplate that eventuality only after we have made an honest effort to understand whether discrepancies may have arisen from *other* causes, be they trivial or interesting. In this case, the cause was trivial: a significantly biased allocation of the face/label combinations used as stimuli. If this confound is controlled for in the analyses, the main result of the original study *is* replicated. In the original Italian sample, being fertile raised partnered women’s attraction to single, relative to attached, men (*p* = 0.001). In the (albeit much noisier) American replication sample, it did too (*p* = 0.002).

In both the original and replication studies, the effect was significant for partnered women and not for unpartnered women. However, in the original study the *difference* between partnered and unpartnered women was significant as well (*p* = 0.007), whereas in the replication study it was not (*p* = 0.170). An obvious reason could be that the replication data were simply too noisy for the interaction to emerge. A conceptually more interesting possible reason concerns a variable that was irrelevant in the original sample but relevant and confounded in the replication sample. The role of this variable (self-confidence with men) was unpredicted, hence this finding should be interpreted as exploratory—a potential factor to track in future research. In the replication but not in the original sample, being partnered strongly covaried with feeling self-confident with men, and feeling self-confident with men was confounded with both face/label allocation and conception risk. Controlling for self-confidence with men replicated the original significant difference between partnered and unpartnered women. The notion that self-confidence with men could play a role is far from counterintuitive: lack of self-confidence may decrease a woman’s perceived chances of success in pursuing an extrapair man, or increase her fear that pursuing an extrapair man could endanger her relationship with her current partner. Still, the effect of fertility on partnered women’s preferences for singles was also significant overall, fully replicating the original finding even if the unanticipated differences in self-confidence between participants are *not* taken into account.

It is important to note that, in principle, the failure to consider the peculiar distribution of women’s self-confidence in the American sample might have hampered replicability entirely, and we would be none the wiser. And yet, the original paper could not possibly have alerted future replicators about the importance of this variable: self-confidence with men was strongly associated with having a partner (and again, confounded with album) in the replication sample, but was unrelated to it (and unconfounded) in the original sample.

## Frequently Asked Questions

In the spirit of open debate I report, with his consent and nearly verbatim, some critical comments made by Ruben Arslan in a signed review of a previous version of this paper. Other readers might easily entertain similar doubts; they sound reasonable but are, I will argue, misplaced. I respond to them here.

*1. The replication data produce the same result as the original data only when two post-hoc-plausible decisions are made. This is a perfect illustration of the problems that led to the reproducibility crisis in psychology. Adjusting for the imbalance in conditions is reasonable, but that alone does not turn the effect significant.*

The original study’s major finding was the effect of cycle on partnered women’s preference for single over attached men. Adjusting for the imbalance in conditions *is* enough to replicate it. Thus, a more appropriate conclusion is that the significant effect for partnered women was replicated, and (although an effect emerged only for partnered and not for unpartnered women) the significant *difference* between partnered and unpartnered women was not.

One may stop there and learn nothing; or wonder why, and perhaps learn something (see also Stroebe and Strack, 2014; Van Bavel et al., 2016; Penders and Janssens, 2018). Here the failure of the *p* = 0.170 interaction to attain significance may very well have been due to the low power of a messy study, or even simply to basic sampling error and random measurement error (Stanley and Spence, 2014), but suppose for the sake of argument that there is a “real” difference between the original and the replication results. To move on, we must look at the data. Self-confidence with men was distributed differently in the original and replication samples, and (only) in the latter it was confounded with partnership status, conception risk, and face/label combinations. I looked exclusively at self-confidence with men because it happened to be the only question that was presented identically to all women. Yet if there had been 10 such theoretically meaningful questions, and one investigated them all to identify those potentially responsible for the difference between the outcomes of the original and replication studies, that would be perfectly rational: what I would ask is that this is done in the open and that the new “findings” are explicitly treated as exploratory. With their help, we may work out better hypotheses, to be tested in future—possibly, preregistered—studies.

*2. The author’s reaction to a nonreplication of her work is to double down on her initial interpretation and reanalyze the data following the Bem advice that has become known as a recipe for overfitting.*

My reaction to a nonreplication of my work is not to prove that my interpretation was correct or my findings “true” (I am in no position to know whether they are), but to understand why the original and replication studies produced different results. Until the day when this attempt to understand is expected from who has failed to replicate—and replication studies and datasets are examined for obvious confounds as scrupulously as original studies and datasets should—the burden is going to fall, alas, on the original authors.

Of Bem’s (1987) otherwise unfortunate recommendations, one should not be dismissed with the rest and it is the only one I have followed here: look at the data. The nonreplication has prompted me, before all else, to reanalyze *my* old data to check to which extent the results I obtained depended on the analytic choices I made (as per Steegen et al., 2016). Even though the multiverse of possible choices in such a complex study is inevitably too large for present-day comfort, the original results have turned out to be remarkably robust—and this includes plausible alternative classifications of participants into high- and low-fertility groups. In fact, the results came out stronger using the stricter window (days 10–15) defined as “peak fertility” in Gildersleeve et al.’s (2014) meta-analysis.

Incidentally, I am not inclined to take this finding as additional evidence for the ovulatory shift hypothesis. Psychology studies typically rely upon relatively small samples. If indeed the fertile window falls entirely between days 10 and 17 in only 30% of women (Wilcox et al., 2000), its average position may be expected to vary even widely from one small sample to the next. Thus, finding the strongest effect for days 10–15 is not necessarily more persuasive than finding it for days 7–14 or 8–20.

*3. Without realizing it, the author is doing what she criticizes herself: wander through the garden of forking paths.*

The “garden of forking paths” (Gelman and Loken, 2013) refers to the idea that the route toward statistical significance appears predetermined but is in fact the result of a hidden chain of choices that, albeit defensible and made in good faith, are arbitrary. Alternative data can lead to equally reasonable alternative analyses and equally reasonable ways to support the research hypothesis; “significant” patterns are thus perpetually revealed in what is actually noise. Very true. But because making reasonable choices cannot be avoided, the only moral is that we should not be so sure of our findings.

In this paper I have not tried out different data-cleaning, data-coding, and/or data-analytic alternatives in the attempt to produce the original results from the replication dataset. And as far as I am aware, I have not made any “reasonable choices” that had not been made in the original study either: I merely checked no obvious confounds had been introduced. I found at least a major one, concerning stimulus allocation, in the replication sample (but not in the original sample). Controlling for it in the analysis revealed a significant cycle shift in preference for single men among partnered women; this shift was in the same direction as reported by Bressan and Stranieri (2008) and replicated their main result.

For exploration purposes, I also showed that controlling for another likely confound (self-confidence with men) replicates their secondary result too. It should be clear that labeling this as a confound rests on the assumption that a woman’s self-confidence with men affects her probability to become involved in an extrapair liason: a reasonable assumption in a world of alternative reasonable assumptions—one path in the garden of forking paths. Even worse, one taken in the context of the replication study, a dismally noisy and confounded dataset.

Of course, *both* the original result and its replication could just be side effects of phenomena unrelated to the hypothesis; or—far from impossible, considering how imprecise all these measures, most notably fertility ones (Wilcox et al., 2000), are bound to be—they might be plain noise themselves. The original findings of Amodio et al. (2008), Campbell and Robert (2008), Monin et al. (2008), and Schnall et al. (2008)—and even the findings “successfully” replicated by the Reproducibility Project, for that matter—might all turn out to be false positives. Yet this is irrelevant to the point I wish to make: let us check whether our data contain obvious confounds before doing anything with them. And if openly controlling for a *demonstrated* confound (not simply a *possible* or *plausible* confound) is now to be considered as a discretionary, arbitrary choice in data analysis, well, we should think again.

## Coda: Let us Hunt for Artifacts

Undisclosed flexibility in data coding and analysis may be responsible for the better part of the replication “crisis” in psychological (and nonpsychological: Begley and Ellis, 2012; Camerer et al., 2016) research (Simmons et al., 2011). Bem (1987) famously encouraged the beginning social scientist to examine the data from every angle; and then, to “cut and polish” the dataset and “craft the best setting for it” as though it were a jewel. Advice of that description tends now to be less popular than it once was. We have become aware that decisions as minor as whether or not to remove outliers, or include a certain factor in the analysis, are capable of swaying a study’s results to the extent of reversing statistical significance (Steegen et al., 2016). Typically, such choices are not portrayed as arbitrary and any alternatives remain hidden. No discretionary paths were taken here. All coding, processing, and analytic choices were identical to those in Bressan and Stranieri (2008); the replication datasets were analyzed as they were provided by the Reproducibility Project. Only transparently verifiable errors and confounds were, respectively, corrected and controlled for. In the interest of cross-validation, each new analysis run on the replication data was repeated identically on the original data. All outcomes are reported.

The potential role played by methodological or statistical problems in purported failures to replicate has been voiced before (see Zwaan et al., 2018), although it appears that the original authors’ viewpoints struggle to be heard, confined as they often are to blogs and comment sections. The particular case I have dissected here stands out from the rest in that the major confound could not only be identified but also controlled for—revealing results that were similar to those reported in the original study. More often (as in all the other cases I have illustrated), methodological confounds are impossible to control after the data have been collected, but that is exactly the stage when they tend to be found. Hardly everything that could possibly go wrong with a study can be predicted ahead of time, even when the study has been meticulously laid out and has received all necessary blessings. And although data are expected to be scrutinized by both authors and peer reviewers before being added to the published record, it appears that no after-the-fact quality control is required of data collected for purpose of replication (see also Schnall, 2014a,b). After-the-fact *any*thing is bunched together with questionable research practices. Johnson et al. (2014) dismissed Schnall’s (2014a) exposure of a ceiling effect in their data as “hunting for artifacts.” But hunting for artifacts is precisely what we should all do before taking our data seriously. If our data *are* the result of artifacts, they carry no evidentiary value; we should dispose of them (well, store them away) and start afresh.

One is left to wonder how many replications “fail” (and also, of course, how many original studies “succeed”) solely because one has not bothered to look carefully at the data. No help will be forthcoming from preregistrations and similar declarations of intent—because whether a replication has failed owing to an unpredictable stimulus misallocation, or an accidental recruitment quirk, or an unexpected sample difference, can be established only *post hoc*. None of us gets a kick out of establishing things *post hoc*. Still, if we are really curious about the truth—as opposed to just craving to prove a point—it might be best to have a good look at the data; yes, to examine them (in full public view) from every angle.

## Data Availability

All data, materials, analysis scripts and outputs, along with the Supplementary Material file, are publicly available via the Open Science Framework and can be accessed at https://osf.io/amrwu.

## Author Contributions

The author confirms being the sole contributor of this work and has approved it for publication.

### Conflict of Interest Statement

The author declares that the research was conducted in the absence of any commercial or financial relationships that could be construed as a potential conflict of interest.

## Footnotes

^1^Relative to the original study, the numerosity of the group of participants who should show the effect—partnered women—was only marginally higher in the online study and actually *lower* in the lab study (see Figure 1), which falls short of qualifying either study as a high-powered replication (e.g., Simonsohn, 2015). Any power calculations the replication team performed prior to data collection (based on the effect size obtained in the original study, a method that leads to inadequate statistical power *per se*: Maxwell et al., 2015; Simonsohn, 2015) were made pointless by the introduction of random and systematic noise that was absent in the original study—not just regular noise, alas, but participants who “forgot to read labels” and biased assignments of stimuli. To ensure sufficient power, the data of the two studies were therefore analyzed together, with type of study (lab replication, online replication) as a factor.

^2^Owing to the different distribution of responses, median splits—i.e., those that created two groups as close as possible in numerosity—turned out to be different in the original and replication samples. Both replication samples: responses 1-2-3 vs. 4-5. Original sample: responses 1-2 vs. 3-4 (nobody chose the value 5). Note that using subdivisions other than the median split would result in extremely unequal cell sizes. Overall, for example, the middle point of the scale (response 3) was chosen by nearly half of the participants; responses 1 and 5 were chosen by only 3% of, respectively, partnered and unpartnered women.

^3^The only results not reported in Figure 1 are the main and interaction effects of type of study and album, which have no bearing on the ovulatory shift hypothesis; they can be found in the analysis outputs (Data Availability).

^4^The significant main effect of fertility on preference for single men corresponds to the significant interaction between conception risk and man’s availability in Bressan and Stranieri’s (2008) repeated-measures ANOVA. Following ANOVA reporting conventions, this effect was not originally reported because it was further qualified by the interaction with participant’s partnership status.
